# Portable BLAST-like algorithm library and its implementations for command line, Python, and R

**DOI:** 10.1371/journal.pone.0289693

**Published:** 2023-11-30

**Authors:** Steven Schmid, Aditya Jeevannavar, Timothy R. Julian, Manu Tamminen

**Affiliations:** 1 hakuna AG, Zürich, Switzerland; 2 Department of Biology, University of Turku, Turku, Finland; 3 Department of Environmental Microbiology, Swiss Federal Institute of Aquatic Science and Technology (Eawag), Dübendorf, Switzerland; Bangladesh University of Engineering and Technology, BANGLADESH

## Abstract

Basic local-alignment search tool (BLAST) is a versatile and commonly used sequence analysis tool in bioinformatics. BLAST permits fast and flexible sequence similarity searches across nucleotide and amino acid sequences, leading to diverse applications such as protein domain identification, orthology searches, and phylogenetic annotation. Most BLAST implementations are command line tools which produce output as comma-separated values files. However, a portable, modular and embeddable implementation of a BLAST-like algorithm, is still missing from our toolbox. Here we present nsearch, a command line tool and C++11 library which provides BLAST-like functionality that can easily be embedded in any application. As an example of this portability we present Blaster which leverages nsearch to provide native BLAST-like functionality for the R programming language, as well as npysearch which provides similar functionality for Python. These packages permit embedding BLAST-like functionality into larger frameworks such as Shiny or Django applications. Benchmarks show that nsearch, npysearch, and Blaster are comparable in speed and accuracy to other commonly used modern BLAST implementations such as VSEARCH and BLAST+. We envision similar implementations of nsearch for other languages commonly used in data science such as Julia to facilitate sequence similarity comparisons. Nsearch, Blaster and npysearch are free to use under the BSD 3.0 license and available on Github Conda, CRAN (Blaster) and PyPi (npysearch).

## Introduction

Basic local-alignment search tool (BLAST) is a versatile sequence analysis algorithm which permits fast sequence similarity searches across DNA, RNA, and amino acid sequences. BLAST searches are used in diverse applications such as sequence similarity searching [1], protein domain identification [2], orthology searches, [3] and phylogenetic annotation [4].

Common implementations of BLAST include the popular web interface provided by NCBI [1] as well as standalone command-line tools such as BLAST+ [5], usearch, [6] and VSEARCH [7]. In a typical use case of BLAST through the NCBI web interface, the BLAST input is submitted through a web form and the results are visually inspected using a web browser. In high-performance applications, BLAST is typically used as a Unix command line application and the output is saved into a comma-separated values table, to be processed further using programming languages such as R or Python. To facilitate this transport of data between the command line application and these programming languages, several wrappers are available such as rBLAST [8] or metablastr [9]. These wrappers require separate installation of the command line application which limits the portability and modular use of BLAST in larger processing pipelines.

To provide a portable BLAST-like algorithm, we present here nsearch which is a dependency-free C++11 library and command line application. C++ is suitable for high-performance applications and is supported as an extension language by several other programming languages. As concrete examples of the portability of nsearch, we introduce Blaster which implements a BLAST-like algorithm in R programming language [10] by embedding nsearch through Rcpp [11], and npysearch which provides similar implementation of nsearch on Python programming language [12] through pybind11 [13]. We envision similar implementations of nsearch for other languages commonly used in data science such as Julia. nsearch is light-weight, has no external dependencies, and currently supports similarity searches of DNA, RNA, and amino acid sequences.

## Materials and methods

### Alphabets

To distinguish between different types of sequences, nsearch uses the concept of alphabets. Alphabets define how biological sequences and their letters are treated. nsearch currently supports two alphabets: nucleic acids (DNA and RNA) and amino acids (defaulting to BLOSUM62 scoring matrix [14]), which are implemented via C++ templates. This enables straightforward implementation of custom alphabets or different scoring schemes for future releases.

### nsearch database indexing and searching

nsearch incorporates database searching inspired by usearch [6] which is based on the idea that a few good candidate sequences are sufficient for most sequence similarity searches. Candidate sequences are selected from a database based on the number of unique words or k-mers (the collection of which is designated *U*) they share and are checked in decreasing order of *U* for their sequence similarity. If the similarity exceeds a user-defined threshold, the candidate is considered a hit. If the similarity is below the threshold, the candidate is considered a reject and the next best candidate is checked, until the maximum number of allowed rejects for this query has been reached.

Database searching in nsearch starts with indexing during which the database sequences are stored in lookup tables. The search algorithm will use the lookup tables to quickly identify shared k-mers across all database sequences. By default, nsearch uses *k* = 8 for the DNA alphabet and *k* = 5 for the Protein alphabet. Higher *k* leads in general to faster search run time at the price of reduced sensitivity [15]. Within nsearch, each k-mer is transformed into a compact binary format (32-bit unsigned integer), described by the BitMapPolicy of the alphabet (e.g. 2 bits are used to encode all DNA nucleotides).

K-mers which contain at least one ambiguous residue are mapped into a special k-mer called AmbiguousKmer. Storing each possible realization of an ambiguous k-mer is infeasible due to exponential growth and corresponding burden on memory consumption and decrease in lookup efficiency. For this reason, regions containing ambiguous nucleotides are discarded in nsearch during database indexing.

Based on the principle that similar sequences tend to have more k-mers in common [16], the search algorithm uses the indexed database to search for candidate sequences by counting the number of unique k-mers (*U*) they share with the query sequence. The sorted list of high-scoring candidates is then checked in decreasing order of *U*: the database sequence which shares the highest number of unique k-mers with the query sequence is checked first. For this purpose, the pairwise sequence alignment is determined. The sequence alignment is used to calculate the sequence identity, which in turn is used to determine whether the candidate is a hit.

The sequence alignment algorithm determines HSPs (high-scoring segment pairs) by employing a seed-and-extend strategy [17]. The HSPs are subsequently joined into a chain in a greedy manner: The highest scoring HSP is combined with the next-best non- overlapping HSP, until there are no HSPs left to join. The missing alignments between the HSPs are determined with banded dynamic programming [18].

### nsearch R and Python interfaces: Blaster and npysearch

Blaster exposes the nsearch BLAST-like functionality to R through Rcpp [11] and to Python through pybind11 [13]. These implementations expose two functions: *read_fasta* which is a utility function for parsing Fasta files into data frames or dictionaries (for R and Python, respectively), and *blast* which is an interface to the underlying nsearch BLAST implementation. *Blast* accepts the following arguments: maximum accepted results, maximum rejected results, minimum hit identity, alphabet, the searched strands, and an option to save the results into a temporary file.

### Benchmarking

The benchmark data set was created using the Rfam 13.0 database [19]. The Rfam database is an annotated collection of non-coding RNA (ncRNA) families and is based on multiple sequence alignments. The ncRNAs are divided into families based on evolution from a common ancestor and the sequences are annotated with taxonomic identity of the host organism. The division into families, the taxonomic annotation, as well as the high sequence diversity permits using Rfam to benchmark the accuracy of the searches of nsearch and other tools.

The following search settings were used: (1) report one hit (which has at least a 75% sequence identity between query and database sequence), (2) terminate after 8 rejects (high scoring candidates which have below 75% sequence identity), (3) use all available CPU cores, (4) no masking.

The following hardware was used: Macbook Pro M1, 2020, 16 GB Memory, running macOS Monterey 12.5.

## Results

For every Rfam family which contains 2 or more sequences, one sequence was selected randomly for the query set (resulting in 2,085 sequences), with the remainder in the reference database (380,796 sequences). Given the high sequence diversity of the Rfam database, the search algorithm should ideally recoved a sequence from the database corresponding to the query sequence. The accuracy of the search was therefore calculated as the proportion of the matches where the database match corresponded to the query sequence. nsearch performance was directly comparable with VSEARCH and BLAST+, both in run time and accuracy (Table 1). The code and data for running the benchmarks is available at https://zenodo.org/record/4804623.

**Table 1:** Search performance comparison of nsearch to other tools. Mean run time is calculated from 10 replicates, using a query set of 2,085 sequences against a database of 380,796 sequences from Rfam. Accuracy is calculated as the percentage of matches between the query and database target sequences.

Software	Mean run time (s)	Accuracy	Notes
BLAST+	1.4	98.1%	The legacy BLAST algorithm
VSEARCH	0.88	98.7%	Another, non-modular implementation of the BLAST algorithm [7]
nsearch	1.52	98.3%	Modular implementation of the BLAST algorithm (this study)
Blaster	2.63	98.3%	Implementation of nsearch on R using Rcpp (this study)
npysearch	1.43	98.3%	Implementation of nsearch on Python using pybind11 (this study)

## Discussion

nsearch is an efficient, dependency-free implementation of a BLAST-like algorithm, written in C++11. Benchmarking nsearch against the commonly used general purpose tools, VSEARCH [7] and BLAST+ [5], demonstrates that nsearch can compete in run time and accuracy with comparable state-of-the-art tools. To demonstrate the portability of nsearch, we present here Blaster and npysearch, implementations of a BLAST-like algorithm on R and Python programming languages, respectively, powered by nsearch. This portability will permit implementations of nsearch into other languages commonly used in data science such as Julia or Ruby, and into larger software frameworks such as Django [20] or Shiny [21] applications. The source code for nsearch, npysearch and Blaster is released under the BSD-3 license and available on Github at https://github.com/stevschmid/nsearch, https://github.com/tamminenlab/npysearch and https://github.com/tamminenlab/blaster, respectively. Furthermore, nsearch, Blaster, and npysearch are available on Conda-forge at https://anaconda.org/conda-forge/nsearch, https://anaconda.org/conda-forge/r-blaster and https://anaconda.org/conda-forge/npysearch, respectively. Blaster is also available on CRAN at https://CRAN.R-project.org/package=blaster and npysearch on PyPi at https://pypi.org/project/npysearch/. The datasets generated and analysed during the current study are available at Zenodo, https://zenodo.org/record/4804623.
